# Which Moral Foundations Predict Willingness to Make Lifestyle Changes to Avert Climate Change in the *USA*?

**DOI:** 10.1371/journal.pone.0163852

**Published:** 2016-10-19

**Authors:** Janis L. Dickinson, Poppy McLeod, Robert Bloomfield, Shorna Allred

**Affiliations:** 1 Department of Natural Resources, Cornell University, Ithaca, New York, United States of America; 2 Department of Communication, Cornell University, Ithaca, New York, United States of America; 3 Samuel Curtis Johnson Graduate School of Management, Cornell University, Ithaca, New York, United States of America; Universidad Miguel Hernandez de Elche, SPAIN

## Abstract

Jonathan Haidt’s Moral Foundations Theory identifies five moral axes that can influence human motivation to take action on vital problems like climate change. The theory focuses on five moral foundations, including compassion, fairness, purity, authority, and ingroup loyalty; these have been found to differ between liberals and conservatives as well as Democrats and Republicans. Here we show, based on the Cornell National Social Survey (USA), that valuations of compassion and fairness were strong, positive predictors of willingness to act on climate change, whereas purity had a non-significant tendency in the positive direction (*p =* 0.07). Ingroup loyalty and authority were not supported as important predictor variables using model selection ($\Delta \underline{\underline{\text{AICc}}}$). Compassion and fairness were more highly valued by liberals, whereas purity, authority, and in-group loyalty were more highly valued by conservatives. As in previous studies, participants who were younger, more liberal, and reported greater belief in climate change, also showed increased willingness to act on climate change. Our research supports the potential importance of moral foundations as drivers of intentions with respect to climate change action, and suggests that compassion, fairness, and to a lesser extent, purity, are potential moral pathways for personal action on climate change in the *USA*.

## Introduction

Working to reduce carbon emissions requires worldwide cooperation and the United States has lagged behind in this effort compared to the developed nations of Europe [1]. Although top-down solutions are critical, large-scale behavior change can be a starting point for significant climate change action, especially if it helps to generate changes in governance. If appeals to moral values tend to be successful at eliciting and sustaining behavior change [2], which is still an open question, then exploring which values are associated with willingness to act on climate change can lead to new insights into the design of more effective communication campaigns, as well as education and citizen science efforts (e.g., YardMap.org). Here we use Moral Foundations Theory (MFT) [3] to explore which of five categories of moral judgments predict willingness to take personal action on climate change in the *USA*.

The dim view of human potential for collective action that arose in the mid-nineteenth century [4,5] was revolutionized in the past few decades as theoreticians began to recognize that social costs and benefits of cooperative acts could be as important as the material costs and benefits [6,7]. The theoretical result is that collective action is more likely when individuals can choose their cooperation partners, when their actions and reputations are visible to others, and when cooperative social norms and benchmarks are made salient [8–10]. Including the potential for social rewards and sanctions in game theoretic models and empirical studies of environmental behavior has already painted a more optimistic picture of the potential for humans to avoid the tragedy of the commons [11] than those originally put forth by Olson [5] and Hardin [4]. Increased understanding of social costs and benefits driving cooperation [8] provides a theoretical basis for inclusion of normative beliefs and subjective social norms in models of behavior change, like the theory of planned behavior [12], but even with these new insights, our ability to design effective programs that move large groups of people towards new kinds of energy use and lower levels of consumption is still limited.

Although game theory addresses the ultimate, evolutionary causes of behavior, a broad array of proximate, psychological and cognitive mechanisms potentially influence the willingness of individuals and groups to act on difficult problems like climate change. Response to climate change can have existential [13], moral [14,15,16], social [10], and pragmatic components [17–19]. This complexity means that the relationship between attitudes, intentions, and behavior is not straightforward [19]. Even when we believe climate change is real and caused by humans, heuristics and cognitive biases can hinder our ability to act [20,21,22]. Understanding which behaviors are most effective also matters, as do sense of self- or group-efficacy and contextual factors [19]. People who exhibit environmental concern do not always know which behaviors help with a particular environmental problem, for example, they may underestimate the effect of purchase choice on carbon emissions [19]. Individual differences and culture shape how people respond through their effects on self-construal, how people see themselves in relation to others and the natural world [23]. Proenvironmental attitudes tend to be associated with more ecocentric and less anthropocentric values [17]. People who define themselves as having an implicit connection to others (meta-personal self-construal) are most likely to express environmental concern and take action [23].

Culture also influences social norms and people’s responses to descriptive social norms. Experiments based on community-based social marketing [24], especially those using descriptive social norms [10], indicate that people can be nudged towards increased energy efficiency simply by receiving information about what others do. This kind of information is particularly powerful when it is hyper-local [10]. As these diverse studies indicate, there is no single, clear path to climate change action. Further, we currently know little about how these different drivers of attitudes, intentions, and behavior interact with each other to determine behavioral outcomes.

Even more controversial is the importance of moral values to conservation intentions and action [25]. Although values have been examined as drivers of attitudes, intentions, and behavior in a variety of conservation contexts, the focus has largely been on ecocentric/anthropocentric values [17]. These and other values are highly contextualized and can be thwarted or repressed by exposure to utilitarian, economic concerns [15,16]. What is less well understood is how moral judgments that operate across a much broader array of contexts influence climate change intentions and action; these overarching values may have important associations with climate change beliefs, attitudes, intentions, and behavior, just as they do with attitudes towards gun control, flag burning, and immigration [26].

Moral Foundations Theory (MFT) and its associated empirical work have provided validated measures of the moral profiles of individuals, generating a robust framework for understanding how moral judgments shape human attitudes, intentions, and behavior [27]. MFT is based on moral pluralism, and defines generic foundations of moral consideration by measuring how people in extant populations make moral decisions, rather than prescribing *a priori* what is moral and what is not within a particular context [28]. In the process, it has introduced the idea that people vary in the extent to which they rank five different classes of moral values: care/harm (compassion/harming); fairness/cheating; ingroup-loyalty/betrayal; authority/subversion; and sanctity/degradation (purity). The generality of these moral axes to diverse situations and their strong association with attitudes that divide liberals and conservatives in the *USA*, potentially allow for increased understanding of moral drivers of climate change action. Because a single topic like climate change can evoke multiple axes at once, people will have different moral profiles, making it difficult to predict what they are willing to do based on one or two axes alone. Including all five axes in the same model can provide an understanding of how generic moral values influence behavioral intentions. In this study we move beyond attitudes to determine which of the five moral foundations predict participants’ self-reported willingness to make lifestyle changes to reduce their personal carbon footprints. We refer to this as “willingness to act.”

Although moral foundations are properties of individuals, MFT offers insights into how moral diversity influences the intentions and behaviors of groups [29]. Interestingly, the five moral axes map to different mechanisms of collective action. Expressions of compassion and fairness can influence whether we are seen as (or feel like) good cooperators; in this regard, they should function largely to ensure cooperation based on reputational mechanisms [11]. In contrast, ingroup-loyalty, purity, and authority serve a binding function, influencing the ability of group members to recognize each other, develop a group identity, and establish a set of rules that enable them to coordinate their actions, often doing so at the expense of autonomy and self-expression [29]. Both individualistic and group-binding mechanisms can potentially influence collective behavior.

In the USA, foundational work in MFT has established differences in valuation of the five moral axes between liberals and conservatives [30]. Conservatives show lower valuation of compassion and fairness, and higher valuation of purity, authority, and in-group loyalty than do liberals. This suggests that ideology binds people with similar moral foundations together, but where climate change is concerned, ethical discussions have focused mainly on nonharming and fairness, assuming that the other moral axes (those most prized by conservatives) are not relevant [31]. Viewed in terms of nonharming and fairness alone, climate change is the “perfect moral storm” in demanding that we weigh current wellbeing against harm to future generations [32].

Recent evidence indicates that framings that evoke compassion or purity can independently increase pro-environmental attitudes [33]. In one study, evoking compassion for nature led to increases in pro-environmental intentions [34]. But how do moral foundations, measured as valuation of the five moral axes, exert a combined influence in the absence of any particular moral framing? In order to address this question, we used data from the Cornell National Social Survey and included as predictor variables all five moral axes, controlling for ideology, belief in climate change, and other factors that are likely to influence climate change attitudes, intentions, or behavior. When analyzed independently, valuations of fairness and compassion were positively associated with support for climate change mitigation policy in Australia, whereas valuation of ingroup loyalty, authority, and purity were negatively associated with policy support [35]. To our knowledge this is the first study to investigate which moral foundations are associated with willingness to take personal action on climate change. Our research is based on a national survey of the American public, and uses a moral diversity approach, testing for all five moral axes at once, alongside other established predictor variables, to investigate moral pathways to climate change action.

## Methods

### Ethics Statement and description of survey

This research is based on questions included in the Cornell National Social Survey (CNSS) of 1,000 adults contacted by telephone in 2014. The phone sample, provided by Marketing Systems Group, was a Random Digit Dial (RDD) list drawn from the continental United States, and included cell phones. The sample selection procedure ensured that every household with a phone had an equal chance to be contacted and, once contacted, every adult in the household had an equal chance of being included in the study. Telephone data collection began on August 9, 2014, and was completed October 26, 2014. All interviews were conducted in English using a Computer Assisted Telephone Interviewing (CATI) software system. Questions for the survey (N = 64) were submitted by researchers at Cornell and selected by the SRI Advisory Board.

The CNSS was conducted with approval of Cornell University’s Internal Review Board (approval #1402004459). Informed consent was obtained as follows. The interviewer first read the statement: “Cornell University is conducting an annual study on people's opinions about education, health care, the environment, and several other important issues. Participating in this survey is a great way to help policy makers understand how people feel about these topics so it's very important that we get your input.” The interviewer then read the following statement about confidentiality: “I want to assure you that all the information you give will be kept completely confidential and that none of it will be released in any way that would permit your identification. Your participation in this study is, of course, voluntary. If there is any question that you would prefer not to answer, just tell me and we will go on to the next question.” If a respondent consented to participate in the study at this stage, the survey program recorded a response of ‘1’ and proceeded with asking questions.

### Data analysis methods

Our study combined data from questions submitted to the survey by three different research groups. This led to an opportunity to investigate the relationship between Haidt’s moral axes and self-reported willingness to take personal action on climate change. The response variable was based on answering the question,” How willing are you to change your current lifestyle in order to reduce your carbon footprint (i.e., to decrease the amount of greenhouse gases you emit)?” (S1 Table). This response variable and the 9 explanatory variables used in this study are described in S1 Table.

Explanatory variables used in the analysis included participants’ valuations of the five moral axes as well as their age, gender, ideology, political party, religiosity, belief in climate change, and level of political activity. Age was considered as a continuous variable, and ranged from 18 to 103. Gender was coded as male (0) or female (1) by the interviewer. We turned answers to missing values if participants refused to answer, or answered “don’t know”, or if they answered “other party” for political party. This was necessary because these answers could not be placed on a scale for analysis as a numeric fixed effect, nor did they comprise a meaningful fixed effect category for analysis. Questions about belief in climate change and political party were the only ones that elicited more than 7 missing values; belief in climate change had 67 missing values and political party had 15 missing values (S1 Table). We coded missing values as <NA> in Program R version 3.1.3, and eliminated participants’ data if *any* analyzed value was missing. This resulted in a sample size of 915 participants.

The Cornell National Social Survey comprises questions contributed by different investigators. As part of a different study, the statement immediately preceding the question that generated our outcome variable (willingness to take personal action on climate change) was presented to respondents based on random sampling of five different wordings or framings. Preliminary analyses (McLeod and Dickinson, unpublished analyses) showed that these five different framings had no significant effect on the outcome variable analyzed here. This allowed us to use the full data set less the 85 participants for which there were missing values for one or more fields of interest to our analysis.

We used Spearman-rank tests for correlations between ideology or belief in climate change and valuation of the five moral axes (compassion/nonharming, fairness, purity, authority, and in-group loyalty). These correlations were used to determine whether these relationships in our sample were consistent with findings from prior studies.

We used a model selection process based on Akaike’s information criterion to determine which models were best supported [36]. Our response variable violated the distributional assumptions of a generalized linear mixed model in being heavily skewed towards four and five. Because this skew could not be addressed via transformation, we used a multinomial logistic regression model. We collapsed the response variable from five to three numeric factors as follows: we collapsed 1 and 2 into 1, 3 into 2, and 4 and 5 into 3. We did a parallel analysis with the 5-level response variable to verify that the recategorization of the response variable did not alter the main results.

Our model selection process included a null model and nine additional models with 7–12 explanatory variables; we started with the most complete model and removed response variables one at a time. The most complete model had all five of the MFT moral axes as well as seven control variables: age, gender, belief in climate change, ideology (very liberal to very conservative), political party (which was correlated with ideology: rho = .55, N = 915, *p*<0.001), religiosity, and level of political activity (S1 Table). Explanatory variables were removed in the following order: political party, religiosity, level of political activity, authority, and ingroup loyalty. We considered the best-supported model among those with $\Delta \underline{\underline{\text{AICc}}}<2$ (S2 Table) and used significance tests to determine which explanatory variables were important predictors of willingness to act on climate change.

We also tested a model without belief in climate change to determine if the result was biased due to having eliminated 65 people who refused to answer or indicated that they did not know if climate change was happening. Removing the belief question allowed us to include these individuals in the analysis (providing there was no other missing value for analyzed variables) and check for bias in the results.

## Results

Our sample of 1,000 participants was 50.2% male and the mean age was 49.3 ± 0.6 (18–102 years old). 78.1% of participants reported that they believed climate change is happening and most (59.3%) were either willing or very willing to take personal action on climate change. Conservative participants placed a lower value on compassion and fairness, while also placing a higher value on purity, authority, and in-group loyalty than did liberal participants (Table 1); this pattern is consistent with findings from earlier research that focused explicitly on differences in valuation of moral axes between liberals and conservatives [30]. People who believed climate change is happening also placed a higher value on nonharming and fairness and a lower value on purity, authority, and ingroup loyalty than did people who did not believe (Table 1).

**Table 1:** Spearman Rank correlations between ideology or belief in climate change and participants’ valuations of the five moral axes from Moral Foundations Theory (rated as 1: strongly disagree to 6: strongly agree).

Moral Axes	Nonharming	Fairness	In-group loyalty	Authority	Purity
Ideology, 1: strong liberal to 7: strong conservative	-.09 **	-.14 ***	.28 ***	.30 ***	.39 ***
Belief climate change is happening, no = 1, yes = 2	.10 **	.12 ***	-.17 ***	-.14 ***	-.16 ***

Data are rho; *N* is 915 unique individuals for whom there were no missing values for any parameters we analyzed in this study of 1,000 participants in Cornell’s National Telephone Survey.

** p<0.01,

*** p<0.001.

The only supported, predictive model, of those we tested, included compassion, fairness, purity, gender, age, belief in climate change, and ideology as predictor variables. Models that included level of political activity, religiosity, political party, and the moral axes ingroup loyalty and authority were not supported (i.e., $\Delta \underline{\underline{\text{AICc}}}$ was greater than 2). High valuations of compassion and fairness were positively associated with self-reported willingness to make personal lifestyle changes to help avoid climate change and were statistically significant predictor variables; valuation of purity was also included as a positive predictor in the best supported model, but had a significance level (*p* = 0.07) greater than the generally accepted alpha level of 0.05 (Table 2). Belief in climate change was significantly associated with increased willingness to act, and had the largest effect size, whereas political conservatism (ideology) and being older and male were significant negative predictors (Table 2). Explanatory variables that were significant predictors (or not) for the 3-level response variable were also significant predictors (or not) for the 5-level response variable. We also tested the best-supported model without the “belief in climate change” predictor variable, to verify that the sample was not biased by having eliminated 65 people who refused to answer or indicated that they did not know if climate change was happening. This model produced results that matched those from our best-supported model (see online appendix, S3 Table).

**Table 2:** Results of the best-supported multinomial logistic regression model predicting participants’ willingness to make personal lifestyle changes to reduce their personal carbon footprint (3-level multinomial response variable).

Predictor variable	Coefficient ± SE Comparing 1–2	Coefficient ± SE Comparing 1–3
Compassion, 1: strongly disagree to 6: strongly agree	0.07 ± 0.10 *ns*	0.22 ± 0.07 **
Fairness, 1: strongly disagree to 6: strongly agree	0.09 ± 0.09 *ns*	0.16 ± 0.06 **
Purity, 1: strongly disagree to 6: strongly agree	0.13 ± 0.09 *ns*	0.12 ± 0.06 *ns*
Belief in climate change, No = 0 to Yes = 1	0.95 ± 0.33 ***	1.57 ± 0.23 ***
Ideology, 1: strong liberal to 7: strong conservative	-0.12 ± 0.09	-0.36 ± 0.07 ***
Age, years	-0.03 ± 0.01 ***	-0.01 ± 0.01 **
Gender, 0: male, 1: female, as factor	0.50 ± 0.27	0.67 ± 0.20 **

The null model and models that included political party, purity, authority, ingroup loyalty, religiosity, and level of activity as explanatory variables were not supported, having AICc’s above 2 and weight of support ≤ 0.13 (S2 Table). The best-supported model had a weight of 0.82. The multinomial regression tested for a unit-increase in the response variable (willingness to act) from 1–2 and 1–3.

* p<0.05,

** p<0.01,

*** p<0.001,

*ns* = not significant.

## Discussion

Our correlational results support Haidt’s [3] findings that liberals and conservatives exhibit different valuations of the five moral foundations identified by MFT. Moreover, the best supported multinomial model provided support for compassion, fairness, and, to a lesser extent, purity, as positive predictors of willingness to act on climate change, controlling for ideological differences, age, gender, and belief that climate change is happening. Models that included valuation of ingroup loyalty and authority were not supported. Belief that climate change is happening, included as a control variable, was the strongest predictor of willingness to take personal action on climate change. Valuation of compassion and fairness were positively correlated with a liberal ideology in this and prior studies, whereas high valuation of purity, authority, and ingroup loyalty were associated with a more conservative ideology [3].

Our results on climate change intentions (willingness to take personal action on climate change) in the *USA* produced slightly different results and conclusions than seen for climate change attitudes based on a population of 487 adult participants in Australia. Our two studies differ in several important respects. First, the response variable in our study was willingness to take personal action on climate change, whereas the response variable in the Australian study was degree of preference for state action on climate change, which is closer to being an attitude than an intention, even though personal action is an implied outcome of state action. Second, our analysis included all five moral foundations in a single model, whereas the five moral foundations were analyzed separately for the Australian study [35]. Although we found strong support for compassion and fairness as predictors of climate-friendly behavioral intentions, we found weak support for purity as a positive predictor, and no support for authority and ingroup loyalty as negative predictor variables. Our results for compassion and fairness were statistically significant, but the result for purity fell just above the generally accepted significance level of 0.05. In contrast, the Australian study showed that valuations of compassion and fairness were positively associated with preference for state action, whereas valuations of purity, authority, and ingroup loyalty were negatively associated with preference for state action on climate change [35]. In other words, the Australian study seemed to show that valuation of moral axes was associated with attitudes about climate change action in much the way one would expect given the association between political ideology (liberal/conservative) and valuation of the different moral foundations. As the context, method of analysis, and the outcomes variables were different between the two studies, it is unclear whether these differences reflect regional differences in beliefs and context, or instead reflect differences in the outcomes variables, survey design, or statistical methods used in the two studies.

Whereas compassion and fairness are generally associated with nonharming and inequity aversion, respectively, purity encompasses a broader and more complex range of possibilities, such as religiosity, chastity, decency, and disgust [27]. Although these traits are highly correlated [28] it is not clear which has possible connections to climate change and its “impure” effects on the planet. One thought is that the earth’s natural state is perceived as having a kind of purity, an idea that seems consistent with preservationist ideologies and could benefit from further investigation [37]. On the other hand, the wide-ranging interpretations of purity may explain its weak relation to climate change intentions in our study.

Will messages that increase salience of the harmful, inequitable, and impure aspects of climate change elicit increased willingness to act in people in the *USA* who place a high value on compassion, fairness, or purity? We cannot assume so, based on Feinberg and Willer’s [33] findings that framing environmental issues in terms of compassion and purity increased pro-environmental attitudes. Because their study did not match frames to inherent moral valuations, we cannot assume that frame-matching predicts attitudes, intentions, or behavior. Further, it is possible that climate change is a more fraught issue than other environmental challenges. Matching message-framing to people’s moral valuations can either reify pre-existing views and intentions or persuade; this makes the predicted outcome of frame-matching less than straightforward. In a recent study, both conservatives and liberals became more entrenched in their attitudes about environmental issues when exposed to message-framing that matched their pre-existing stance, but only conservatives were “persuaded”, showing attitude shifts when presented with counter-attitudinal messages [38]. Whether such differences in potential for persuasion with regard to attitudes also apply to the effects of moral framing on willingness to act on climate change is as yet unclear.

The moral divide between conservatives and liberals is by no means absolute. Conservatives in Graham et al.’s study [30] of moral values and ideology did not place as high a value on compassion and fairness as did liberals (also corroborated here), and placed a higher value on purity and authority than on compassion and fairness, but the slope of decline in valuation of compassion from very liberal to very conservative was shallow, so it is incorrect to assume that conservatives will not be influenced by appeals to compassion and fairness. Testing appeals to compassion, fairness, and purity separately and in combination, while controlling for valuation of all moral axes, should lead to better understanding of whether and when such appeals are persuasive and whether or which morality-based messages elicit significant support for acting on climate change across the liberal-conservative spectrum.

Although compassion is a moral value, it is also an emotion. Our study did not explicitly evoke the emotion of compassion, but a recent study provides evidence that evoking compassion can be persuasive. Asking people to read a story and think about how it feels to be victimized by climate change, while examining a photo of a suffering two-year-old, generated increased support for government action among conservatives and moderates in the USA, compared to participants who were asked to read the story objectively [39]. The compassion treatment had no effect on liberals, whose support was higher than for the two other groups under both the compassion and objective treatments. The compassion treatment increased belief in both human-caused drought and levels of self-reported compassion, which together explained increased support for government action. The study provides compelling evidence of the persuasive nature of invoking the emotion of compassion; for conservatives support for climate change action in the compassion treatment was more than double that in the objective treatment.

Experiments are the gold standard in many fields, but observational “field studies”, such as we report, are needed to approximate patterns as they manifest with a representative spectrum of participants in unmanipulated environments. Studies of the social psychology of environmental behavior can benefit from the methods of behavioral ecology and animal behavior research, in which lab or field experiments are combined with observational field studies with recognition that each approach has its strengths. In the age of sensors and the Internet, new techniques (including crowdsourcing and computational methods) can be used to better understand behavior in the population at large, opening up new possibilities for understanding the feedbacks between and constraints on attitudes, intentions, and behavior in real world settings. Our results provide support for the possibility that moral values are more important to climate change intentions than previously indicated by Markowitz and Shariff [25], who concluded that “climate change fails to generate strong moral intuitions” and “does not motivate an urgent need for action in the way that other imperatives do.” Moral foundations and the social functions they have served in evolutionary time are no doubt responsible, in part, for the intrinsic rewards associated with the “warm glow” in response to having done the ‘right’ thing with respect to the environment [40]. Although attitudes are often studied, more critical are measures of intentions, behavior change, and support for policy. Experimental and longitudinal, observational studies using MFT are really needed to fully examine the perspective of Crompton [2], who suggested that moral values are more important than we realize, especially with regard to sustained behavioral change and the potential for the U.S. to take action on climate change commensurate with what is happening in other parts of the developed world.

## Supporting Information

S1 Table The explanatory variables and response variables from the survey in the order they were asked. The question about willingness to act (response variable) directly preceded the question about belief in climate change (one of our explanatory variables). There were 32 questions between the question about belief in climate change and the five questions about valuation of moral axes and 14 questions between the latter and the demographic/ideology questions.
(DOCX)

S2 Table Results of model averaging showing the model weights for 4 models and the null model. (DOCX)

S3 Table Results of multinomial model after elimination of the ‘belief in climate change’ predictor variable. This model includes 65 additional participants who did not know whether they believed in climate change or not for a total of 980 participants. The analysis was conducted to look for bias due to not including those participants in the original set of models. All predictor variables significant/not significant here were significant/not significant in the more inclusive model.
(DOCX)
