# Supplementary material for: Which Moral Foundations Predict Willingness to Make Lifestyle Changes to Avert Climate Change in the USA?
Source: PLoS One. 2016 Oct 19;11(10):e0163852. doi: 10.1371/journal.pone.0163852 (PMC5070873; doi:10.1371/journal.pone.0163852)
Supplement: S1 Table — The question about willingness to act (response variable) directly preceded the question about belief in climate change (one of our explanatory variables). There were 32 questions between the question about belief in climate change and the five questions about valuation of moral axes and 14 questions between the latter and the demographic/ideology questions. (DOCX) [file pone.0163852.s001.docx]

**S1 Table.** **The explanatory variables and response variables from the survey in the order they were asked.**

| Description | Question asked in survey (gray) and scale (white) |
| --- | --- |
| Willingness to act on climate change [Response variable – recoded for multinomial logistic regression as 1 (1 and 2), 2 (3), and 3 (4 and 5)] | How willing are you to change your current lifestyle in order to reduce your carbon footprint (i.e., to decrease the amount of greenhouse gases you emit)? |
|  | 1. Very Unwilling 2. Somewhat Unwilling 3. Neutral 4. Somewhat Willing 5. Very Willing |
| Belief climate change is happening (recoded as 0=No, 1=Yes) | **Do you believe that climate change is happening?** |
|  | 1. No 2. Yes 3. Don’t know (Missing value = 67/1,000, but only 13 in analyzed sample of 189 participants that did not receive framing statement) 4. Refused (Missing value, N = 0) |
| *The following questions were asked in order as listed:*  Now we are going to ask you a series of questions on your personal beliefs. Please let me know if you agree or disagree with these beliefs. | |
| Nonharming | **Compassion for those who are suffering is the most crucial virtue. Would you:** |
|  | 1. Strongly disagree 2. Moderately disagree 3. Slightly disagree 4. Slightly agree 5. Moderately agree 6. Strongly agree |
| Fairness | When the government makes laws, the number one principle should be ensuring that everyone is treated fairly. Would you: |
|  | 1. Strongly disagree 2. Moderately disagree 3. Slightly disagree 4. Slightly agree 5. Moderately agree 6. Strongly agree |
| In-group loyalty | I am proud of my country's history. Would you: |
|  | 1. Strongly disagree 2. Moderately disagree 3. Slightly disagree 4. Slightly agree 5. Moderately agree 6. Strongly agree |
| Authority | Respect for authority is something all children need to learn. Would you: |
|  | 1. Strongly disagree 2. Moderately disagree 3. Slightly disagree 4. Slightly agree 5. Moderately agree 6. Strongly agree |
| Purity | Chastity is an important and valuable virtue. Would you: |
|  | 1. Strongly disagree 2. Moderately disagree 3. Slightly disagree 4. Slightly agree 5. Moderately agree 6. Strongly agree |
| Age | What year were you born? |
|  | <1910-1994>  <r> Refused [Missing value, N = 4] |
| Ideology | When it comes to social issues, do you usually think of yourself as extremely liberal, liberal, slightly liberal, moderate or middle of the road, slightly conservative, conservative, or extremely conservative? |
|  | 1. Extremely liberal 2. Liberal 3. Slightly liberal 4. Moderate or middle of the road 5. Slightly conservative 6. Conservative 7. Extremely conservative   <r> Refused [Missing value, N = 3] |
| Gender | Recorded as male or female by interviewer |
|  | 1. Male 2. Female |
| Political party | Generally speaking, when it comes to political parties in the United States, how would you best describe yourself? |
|  | 1. Strong Democrat 2. Not very strong democrat 3. Independent (close to democrat) 4. Independent (close to neither) 5. Independent (close to republican) 6. Not very strong Republican 7. Strong Republican 8. Other party (specify) [Missing value, N = 29] 9. Refused [Missing value, N = 5] |
| Religiosity: How often attend religious services | Aside from weddings and funerals, how often do you attend religious services: more than once a week, once a week, once or twice a month, a few times a year, seldom or never? |
|  | 1. More than once a week 2. Once a week 3. Once or twice a month 4. A few times a year 5. Seldom 6. Never   <-1> Refused [Missing value, N= 2] |
| Level of political activity | How politically active would you say that you were over the last 12 months? Political action is any action such as voting, signing a petition, attending a public meeting, or protesting, for the purpose of encouraging political or governmental agencies or individuals to take a particular course of action. [No participants answered don’t know or refused to answer.] |
|  | 1. Not at all active 2. Hardly active at all 3. Neutral 4. Moderately active 5. Very active |

The question about willingness to act (response variable) directly preceded the question about belief in climate change (one of our explanatory variables). There were 32 questions between the question about belief in climate change and the five questions about valuation of moral axes and 14 questions between the latter and the demographic/ideology questions.
