# Supplementary material for: Which Moral Foundations Predict Willingness to Make Lifestyle Changes to Avert Climate Change in the USA?
Source: PLoS One. 2016 Oct 19;11(10):e0163852. doi: 10.1371/journal.pone.0163852 (PMC5070873; doi:10.1371/journal.pone.0163852)
Supplement: S2 Table — (DOCX) [file pone.0163852.s002.docx]

**S2 Table.** **Results of model averaging showing the model weights for 6 models and the null model.**

| Explanatory variables included | Delta AICc | Degrees of freedom | Model weight |
| --- | --- | --- | --- |
| Compassion, fairness, purity; ideology; belief climate change is happening; gender; age | 0.0 | 16 | 0.82 |
| Compassion, fairness, purity, ingroup loyalty; ideology; belief climate change is real; gender; age | 3.7 | 18 | 0.13 |
| All five moral axes; ideology; belief climate change is happening; activity; gender; age | 7.4 | 22 | 0.02 |
| All five moral axes; ideology; belief climate change is happening; gender; age | 7.6 | 20 | 0.02 |
| All five moral axes; ideology; political party, belief climate change is happening; religiosity, activity, gender; age | 8.5 | 26 | 0.01 |
| All five moral axes; ideology; belief climate change is happening; religiosity; activity; gender; age | 11.5 | 24 | <0.01 |
| Null model | 185.8 | 2 | <0.01 |
