# Supplementary material for: Which Moral Foundations Predict Willingness to Make Lifestyle Changes to Avert Climate Change in the USA?
Source: PLoS One. 2016 Oct 19;11(10):e0163852. doi: 10.1371/journal.pone.0163852 (PMC5070873; doi:10.1371/journal.pone.0163852)
Supplement: S3 Table — This model includes 65 additional participants who did not know whether they believed in climate change or not for a total of 980 participants. The analysis was conducted to look for bias due to not including those participants in the original set of models. All predictor variables significant/not significant here were significant/not significant in the more inclusive model. (DOCX) [file pone.0163852.s003.docx]

**S3 Table.** **Results of multinomial model after elimination of the belief in climate change predictor variable.**

| **Predictor variable** | **Coefficient ± SE**  **Comparing 1-2** | **Coefficient ± SE**  **Comparing 1-3** |
| --- | --- | --- |
| Compassion: 1, strongly disagree to 6, strongly agree | 0.07 ± 0.09 | 0.23 ± 0.07*** |
| Fairness: 1, strongly disagree to 6, strongly agree | 0.14 ± 0.08 | 0.19 ± 0.06*** |
| Purity: 1, strongly disagree to 6, strongly agree | 0.08 ± 0.08 | 0.08 ± 0.06 |
| Ideology: 1, strong liberal to 7, strong conservative | -0.19 ± 0.09* | -0.44 ± 0.06*** |
| Age: years | -0.03 ± 0.01*** | -0.02 ± 0.00*** |
| Gender: 0, male, 1, female, as factor | 0.49 ± 0.26*** | 0.67 ± 0.18*** |

This model includes 65 additional participants who did not know whether they believed in climate change or not for a total of 980 participants. The analysis was conducted to look for bias due to not including those participants in the original set of models. All predictor variables significant/not significant here were significant/not significant in the model that included people who did not know whether they believed in climate change.

* p<0.05, ** p<0.01, *** p<0.001
